# Conducting Research with Vulnerable Populations: Cautions and Considerations in Interpreting Outliers in Disparities Research

**DOI:** 10.3934/publichealth.2014.1.25

**Published:** 2014-02-09

**Authors:** Salimah H. Meghani, Eeeseung Byun, Jesse Chittams

**Affiliations:** 1 Department of Biobehavioral Health Sciences, NewCourtland Center for Transitions and Health, University of Pennsylvania School of Nursing, 418 Curie Boulevard, Philadelphia, PA 19104, USA; 2 Department of Family Health Care Nursing, University of California San Francisco School of Nursing, 2 Koret Way, San Francisco, CA 94143, USA

**Keywords:** disparities, inequities, disparities research, research methods, cancer pain, African Americans, outliers

## Abstract

Addressing the needs of understudied and vulnerable populations first and foremost necessitate correct application and interpretation of research that is designed to understand sources of disparities in healthcare or health systems outcomes. In this brief research report, we discuss some important concerns and considerations in handling “outliers” when conducting disparities-related research. To illustrate these concerns, we use data from our recently completed study that investigated sources of disparities in cancer pain outcomes between African Americans and Whites with cancer-related pain. A choice-based conjoint (CBC) study was conducted to compare preferences for analgesic treatment for cancer pain between African Americans and Whites. Compared to Whites, African Americans were both disproportionately more likely to make pain treatment decisions based on analgesic side-effects and were more likely to have extreme values for the CBC-elicited utilities for analgesic “side-effects.” Our findings raise conceptual and methodological consideration in handling extreme values when conducting disparities-related research. Extreme values or outliers can be caused by random variations, measurement errors, or true heterogeneity in a clinical phenomenon. The researchers should consider: 1) whether systematic patterns of extreme values exist and 2) if systematic patterns of extreme values are consistent with a clinical pattern (e.g., poor management of cancer pain and side-effects in racial/ethnic subgroups as documented by many previous studies). As may be evident, these considerations are particularly important in health disparities research where extreme values may actually represent a clinical reality, such as unequal treatment or disproportionate burden of symptoms in certain subgroups. Approaches to handling outliers, such as non-parametric analyses, log transforming clinically important extreme values, or removing outliers may represent a missed opportunity in understanding a potentially targetable area of intervention.

## 1. Introduction

Addressing the needs of understudied and vulnerable populations first and foremost necessitate the correct application and interpretation of research that is designed to understand sources of disparities in healthcare or health systems outcomes. In this brief research report, we discuss some important concerns and considerations in handling “outliers” when conducting disparities-related research. To illustrate these concerns, we use data from our recently completed study that investigated sources of disparities in cancer pain outcomes between African Americans and Whites with cancer-related pain.

Undertreatment of pain in the United States has been characterized by the recent Institute of Medicine report as a public health “crisis,” with an accompanying fiscal burden of up to $635 billion annually [1]. Approximately 14 million Americans are living with the diagnoses of cancer and an additional 1.6 million people are diagnosed with cancer each year [2]. While adequate pain management remains a challenge for all cancer patients, African Americans represent a unique group suffering disproportionally as a result of cancer and cancer pain. Compared to Whites, African Americans have higher rates of cancer and co-morbid conditions and are more likely to seek health care in advanced stages of their disease [3]. Despite this, consistent evidence suggests that African American patients have worst cancer pain outcomes of all racial and ethnic groups due to not only inadequate prescription [4]–[8] but also lack of adherence to analgesics even when they are prescribed to them [9],[10]. The reasons for lack of adherence to analgesia, however, have not been fully investigated. To this end, we designed a choice-based conjoint analysis (CBC) experiment to understand the heuristics and salient concerns underlying analgesic treatment decision-making for African Americans and Whites with cancer-related pain.

CBC is a trade-off analysis technique to understand what people value and what drives them to choose one set of alternatives over another when faced with competing choices [11]. By asking individuals to make trade-offs between an important but limited set of attributes, a unique set of values (“part-worth utilities”) can be derived. These part-worth utilities model the underlying latent preference function such that a higher part-worth utility represents a higher value an individual assigns to that attribute [12].

In our study, the construct of interest was preferences for analgesic treatment for cancer pain. Based on pilot work, a randomized-design, computer-assisted CBC experiment was developed using 5 key attributes: type of analgesic; expected pain relief; type of side-effects; severity of side-effects; and out-of-pocket cost (see Meghani, Chittams, Hanlon & et al., 2013, for detailed description of CBC methods) [13]. The relative importance scores (utilities) of each of these 5 attributes were measured on a continuous scale. The main findings were that, on average, African Americans and Whites employed different heuristics in pain treatment decision-making. African Americans were more likely than Whites to make cancer pain treatment decisions based on type of analgesic side-effects (see Table 1).

Pertinent to the present report, we evaluated the CBC utilities statistically to understand if there were any outliers or systematic patterns to the distribution of these salient variables by racial subgroups. An outlier is an observation further away from the rest of the data usually at least 3 standard deviations from the mean on the standardized scale. Outliers and influential points can be caused by random variations, measurement errors or “true heterogeneity” in a phenomenon [14]. As may be evident, for those conducting disparities-related research, it is critical to investigate the “true heterogeneity” hypothesis by investigating any systematic patterns within the distribution of extreme values—this has implications for correct statistical handling of outliers but more importantly for appropriate interpretation of the subgroup data and subsequent intervention/program development.

**Table 1.** CBC Utilities for Analgesic Treatment Decisions For Cancer Pain By Race (*N* = 241).

CBC Attribute	Whites(*N* = 139)	African Americans(*N* = 102)	*p*-values
Pain Relief with	36.71^‡^	26.83^‡^	< 0.001
Analgesics			
Type of Analgesic	19.29^‡^	28.72^‡^	< 0.001
Side-effects			
Severity of Side-effects	18.55^‡^	16.81^‡^	0.225
Type of Analgesic	13.52^‡^	16.66^‡^	0.176
Out of Pocket Cost	11.93^‡^	10.98^‡^	0.355

CBC= Choice-based Conjoint Analysis

## 2. Materials and Method

Participants were recruited from two outpatient oncology clinics of a tertiary academic medical center in Philadelphia. Patients were included in the study if they were self-identified African Americans or Whites, were at least 18 years of age, and had a diagnosis of solid tumor or myeloma, and cancer-related pain. All patients provided informed consent. The study was approved by the institutional review board of the University of Pennsylvania.

The CBC utilities were estimated using Sawtooth Software CBC/HB system [15]. To understand systematic differences in the distribution of outliers between the two groups, we conducted a test for influential points labeling them by respondent's race/ethnicity and compared these values using histograms and box plots as well as checking highest or lowest values. The assessment was conducted in SPSS for Windows, version 20.0 (IBM Corp., NY, USA).

We define an outlier in a set of data to be an observation (or subset of observations) which appears to be inconsistent with the remainder of that set of data. Statistical calculations can answer this question: If the values were all sampled from a Gaussian (“normal”) distribution, what is the chance that one value will be far away from the rest? Thus, a useful way to quantify an extreme value is by the number of standard deviations that a value is from the mean. This statistic applied to the most extreme value in a sample is called the Extreme Studentized Deviate (or ESD) and is defined as follows: max_i=1,..,n_|Y_i_–y|/S, where y is estimated by the sample mean, and S is estimated by the sample standard deviation [16]. The appropriate critical values depend on the sampling distribution of the ESD statistic for samples of size n from a normal distribution. A more general rule of thumb is to consider any observation greater than 3 standard deviations from the mean as a potential outlier.

## 3. Results

The sample size was 241(African Americans = 102; Whites = 139). There was no difference in age between African Americans and Whites (*p* = 0.194). However, African Americans were more likely females (*p* = 0.019), belonged to a lower income bracket (*p* < 0.001), and were less likely to carry private health insurance when compared to Whites (*p* < 0.001; see Table 2).

**Table 2.** Characteristics of study participants by Race (*N* = 241).

Variable	Total (*N* = 241)	African Americans (*N* = 102)	Whites (*N* = 139)	*p*-values†
Mean (SD)				
Age	53.7 (11.0)	52.7 (10.1)	54.5 (11.6)	0.194
Frequency (%)				
Gender				0.019
Male	111 (46)	38 (37)	73 (53)	
Female	130 (54)	64 (63)	66 (47)	
Marital Status				< 0.001
Married	133 (55)	33(32)	100 (72)	
Separated/ Divorced/Widowed	62 (26)	42 (41)	20 (14)	
Never Married	46 (19)	27(27)	19 (14)	
Education				0.011
Elementary	3 (1)	2 (2)	1 (2)	
High School	84 (35)	42 (41)	42 (42)	
College/Trade	117 (49)	51 (50)	66 (51)	
School				
More Than	37 (15)	7 (7)	30 (7)	
College				
Income				< 0.001
< 30, 000	85 (35)	57 (56)	28 (20)	
30–50,000	44 (18)	26 (25)	18 (13)	
50–70,000	41 (17)	13 (13)	28 (20)	
70–90,000	25 (11)	3 (3)	16)	
> 90,000	46 (19)	3 (3)	43 (31)	
Health Insurance				< 0.001
Private	123 (51)	30 (29)	93 (67)	
Medicaid	33 (14)	28 (27)	5 (4)	
Medicare	50 (21)	25 (25)	25 (18)	
Other	34 (14)	19 (19)	15 (10)	

†*p*-values are based on *t*-tests for continuous variables and chi-squared tests for categorical variables.

**Figure 1.**
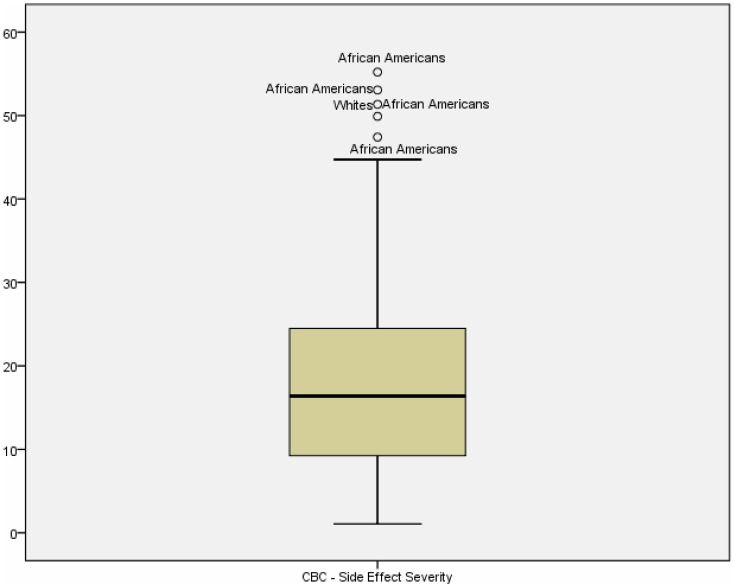
Extreme Observations by Race on the CBC Utility of Side-effect Severity.

CBC utilities had a very clear pattern of extreme values by racial subgroups. For instance, when compared to Whites, African Americans were disproportionately more likely to have extreme values for the utility of “side-effects” (see Figure 1). The systematic patterns of extreme values are consistent with the earlier findings of poor clinical management of pain and side-effects in African Americans [5],[17],[18]. We observed this pattern in other variables (e.g., pain levels and analgesic barriers) that pertained to the phenomenon of interest. These findings raise the need for additional conceptual and methodological considerations in handling outliers in disparities research.

## 4. Discussion

**Figure 2.**
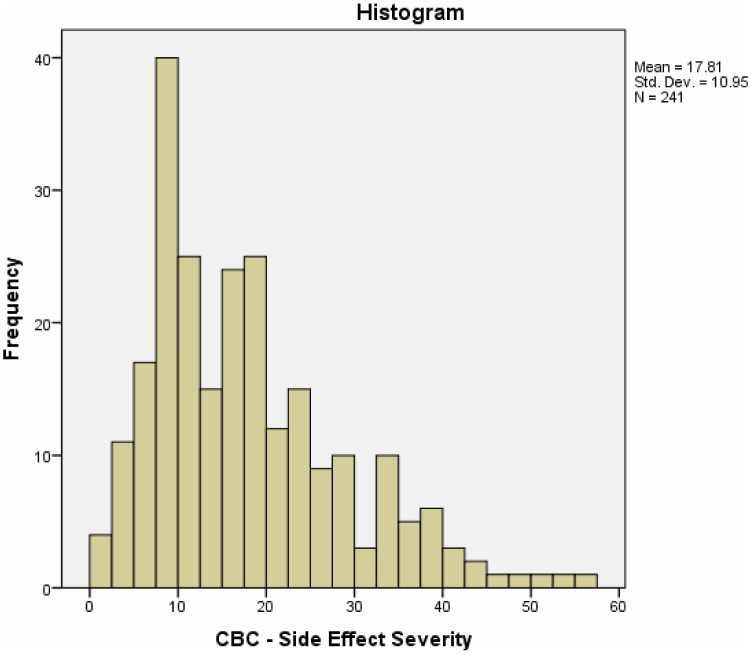
Appearance of a Log Normal (right skewed) Distribution.

An outlier is an observation further away from the rest of the data usually at least 3 standard deviations from the mean on the standardized scale. When outliers on the higher end of the distribution remain in the estimated models, these can result in over inflated means compared to those of models without outliers; thus, resulting in a poor estimate of the central tendency of the population. A histogram plot of the data may reveal the appearance of a log normal (right skewed) distribution (see Figure 2). The variance of a lognormal distribution is a function of the expected mean [19]. For instance, if a subgroup (as in African Americans in our study) has a significantly larger expected mean for a particular lognormal outcome variable, then the researcher may expect the subgroup to have more variability around their mean and more outliers.

It is critical to distinguish whether these outliers are potentially resulting from measurement errors, imply random variations or represent a true heterogeneity in the phenomenon. If outliers are accurate observations that reflect a true heterogeneity in the phenomenon, they could be interesting outliers. Interesting outliers are defined as data that have been regarded as outlying observations but these are not resulting from inaccuracies, such as errors in observations or coding [20]. It may be evident that these considerations are particularly salient in health disparities research where extreme values may actually be representative of a clinical reality, such as unequal treatment or disproportionate burden of symptoms in certain subgroups. Below, we suggest ways to identify and handle outliers in disparities research.

From a statistical perspective, a careful examination of the distribution of the outcome variable of interest could help reveal important racial disparities related outliers. An effective visual method may include box-and-whisker plots or stem and left plots with the race of the extreme observations displayed. Plotting residuals after estimating a model may also identify residuals that appear out of range. It is more likely that observations responsible for these large residuals are outliers [20]. When outliers are detected, it is important to make sure that these are not coding errors (such as a missing data code of 99). If these outliers are not coding errors, in general, estimating a model with or without the outlying cases can be considered. Explaining why these outlying cases are further away from the population of interest rather than removing these outliers from the model would reveal important findings in disparities research.

There may be a mediation effect of extreme values affecting the relationship between race and the outcome. The distribution issue should be addressed before considering the mediation theory. Initially, researchers may examine estimates of the central tendency such as: median, geometric and arithmetic mean, normality tests with or without outliers, or even a *t*-test between racial/ethnic groups. With many common inferential statistical methods, the focus is on measuring the central tendency, area where most of data is centered. A normal distribution assumption is required for a *t*-test when comparing two groups. When this assumption is not met, the impact of outliers and influential data can be diminished by a log transformation of the outcome variable or non-parametric method (e.g., Wilcoxon rank sum test). Since the arithmetic mean is influenced by outliers, it is often replaced by the median or geometric mean in those instances when the data is skewed. Robust approaches, such as generalized estimating equation methods focused on estimating mean population effects, can also be considered to handle outliers [20].

On the other hand, researchers may feel that these outliers represent an important sub-population deserving careful examination to determine if there is something that explains their poor outcome that may be potentially addressed with an intervention. The researchers may actually choose to conduct a case study on these outliers. Thus, removing or log transforming clinically important extreme values or robust approaches may represent a missed opportunity in understanding a potentially targetable area of intervention.
